# The effects of hydroxychloroquine and its promising use in refractory obstetric antiphospholipid syndrome

**DOI:** 10.1007/s00296-023-05457-5

**Published:** 2023-09-23

**Authors:** Juan J. Fierro, Manuela Velásquez-Berrío, Alexandra Ospina, Svenja Henning, Karina de Leeuw, Ángela P. Cadavid J

**Affiliations:** 1 Department of Rheumatology and Clinical Immunology, University Medical Center Groningen, University of Groningen, Hanzeplein 1, 9700RB Groningen, The Netherlands; 2 Grupo Reproducción, Departamento de Microbiología y Parasitología, Facultad de Medicina, Universidad de Antioquia UdeA, Medellín, Colombia

**Keywords:** Hydroxychloroquine, Chloroquine, Antiphospholipid syndrome, Refractory, Pregnancy

## Abstract

Hydroxychloroquine (HCQ) is obtained by hydroxylation of chloroquine (CQ) and the first indication was malaria. Nowadays, HCQ is commonly used in systemic lupus erythematosus (SLE) and rheumatoid arthritis (RA) with favorable results. Antiphospholipid syndrome (APS) is an autoimmune disease characterized by thrombosis and/or pregnancy morbidity and persistent positivity of antiphospholipid antibodies. Around 20–30% of pregnant women with APS develop adverse pregnancy outcomes despite conventional treatment with aspirin and heparin, called refractory obstetric APS. Interestingly, HCQ has shown positive effects on top of the standard of care in some refractory obstetric APS patients. HCQ mechanisms of action in APS comprise its ability to bind sialic acid present in cell membranes, its capacity to block the binding of antiphospholipid antibodies to the cell and the induced increase of pH in extracellular and intracellular compartments. However, the precise mechanisms of HCQ in the specific situation of refractory APS still need to be fully clarified. Therefore, this review summarizes the known modulating effects of HCQ and CQ, their side effects and use in APS and different pathologies to understand the benefit effects and the mechanism of action of HCQ in refractory obstetric APS.

## Introduction

Hydroxychloroquine (HCQ) is a drug obtained by beta hydroxylation of chloroquine (CQ), an alkaloid, similar in structure to the active compounds present in the cinchona bark [1]. Drugs derived from cinchona bark have been used for treating several diseases, mainly malaria, a disease caused by parasites from the *Plasmodium genus* [2]. CQ was first synthesized in 1934 and HCQ in 1960 by chemical modification of CQ [3]. HCQ and CQ have similar pharmacokinetic properties; however, HCQ is more polar and less lipophilic, resulting in less diffusion across biological membranes and thus less toxicity [4].

In the twentieth century, widespread use of quinine by the US Army as a prophylaxis for malaria was accompanied by observations that this drug had positive effects on other diseases such as rheumatoid arthritis (RA) and systemic lupus erythematosus (SLE) [5, 6]. HCQ was implemented in the treatment of these autoimmune diseases afterwards, and it induced favorable effects on diabetes and dyslipidemia in SLE and RA patients [4, 7]. More recently, the use of HCQ and CQ was proposed as a treatment for COVID-19 with controversial results [8].

Antiphospholipid syndrome (APS) is an autoimmune disease characterized by thrombosis (vascular APS) and/or pregnancy morbidity (obstetric APS) and persistent positivity of moderate to high titers of antiphospholipid antibodies (aPL) such as anti-cardiolipin (aCL), anti-ß2 glycoprotein 1 (aß2GP1) or the lupus anticoagulant (LA) in a 12-week interval. APS-related pregnancy morbidity is characterized by consecutive spontaneous abortions before 10th week of gestation, unexplained fetal deaths at or beyond the 10th week of gestation and premature births before the 34th week of gestation because of placental insufficiency [9]. Recently, vascular and obstetric APS have been described as separate entities due to differences in their pathophysiology [10]. Moreover, APS can be primary, in the absence of other autoimmune diseases, or secondary when it is related to another autoimmune diseases, mainly SLE and RA [11].

Around 20–30% of pregnant APS patients develop adverse pregnancy outcomes despite conventional treatment with heparin and low-dose aspirin which is called refractory obstetric APS [12, 13]. The European Alliance of Associations for Rheumatology (EULAR) guidelines recently considered HCQ addition in the first trimester for women with refractory obstetric APS [14]. Adding HCQ to conventional treatment in those patients has led to a 34% live birth achievement increase in the subsequent pregnancy [12]. Indeed, a recent systematic review confirms live birth rate improvement after adding HCQ [15]. However, HCQ effects have been discussed mainly for vascular APS manifestations and the mechanisms of these beneficial effects are still unclear in obstetric APS [16–18]. Therefore, this review describes the effects of HCQ and CQ in APS and different pathologies to propose possible mechanisms of action of HCQ and its promising modulatory effects in refractory obstetric APS.

## Search strategy

A literature electronic search through Medline/PubMed, Scopus, and Directory of Open Access Journals (DOAJ) was carried out for this narrative review. The following keywords were used: ‘Antiphospholipid syndrome’, ‘Obstetric antiphospholipid syndrome’, ‘Refractory’, ‘Hydroxychloroquine’, ‘Chloroquine’, ‘Pregnancy’. We included original articles, narrative and systematic reviews relevant to our review aim. No limits on publication date were placed and publications in languages other than English or Spanish were excluded which was a limitation of our study. All relevant studies until August 2023 were included.

## Pharmacokinetics of HCQ

HCQ is administered orally and is mostly absorbed in the upper intestinal tract with a subsequent bioavailability of 0.7–0.8 and a binding to blood proteins of 40%, of which albumin is the main one [4, 19]. This binding is reversible and creates a balance between the bound drug fraction and the free or pharmacologically active drug fraction. Moreover, HCQ is metabolized in the liver resulting in metabolites like desethylhydroxychloroquine, desethylchloroquine, and bidesethylhydroxychloroquine. These metabolites have a half-life between 30 to 60 days. HCQ is excreted in the urine in a concentration of 15–25% as metabolites and 60% in its unchanged form [19]. Pharmacokinetics of HCQ have been described during pregnancy; higher volume of distribution, higher clearance due to increased glomerular filtration, and increased metabolism by liver enzymes have been described [20].

## Safety of HCQ use

Retinopathy risk is the most important side effect of HCQ. Risk factors for retinopathy are high daily doses (> 5 mg/kg/day), duration of use > 5 years, concomitant tamoxifen treatment, and a decrease in drug elimination due to renal impairment [21, 22]. Furthermore, HCQ is associated with benign corneal deposits, decreased visual acuity, and color vision deficiency. Because of these side effects, the American Academy of Ophthalmology (AAO) recommends a baseline fundus examination to rule out preexisting maculopathy and an extended screening with visual fields and spectral-domain optical coherence tomography in case of abnormalities. Depending on major risk factors, following ophthalmological evaluation should be performed sooner or after five years of use [21, 23].

HCQ overdose toxicity manifestations include cardiovascular shock, hypokalemia, psychosis and seizures due to sodium and potassium channel blockade [24]. Medical care for HCQ overdose is based on supportive treatment [25]. Furthermore, HCQ and CQ can be ineffective or toxic in patients with different genetic conditions [26].

HCQ treatment can be initiated or continued during pregnancy and breastfeeding, and its use has not been associated with adverse pregnancy outcomes [27]. Furthermore, the discontinuation of HCQ therapy before or during pregnancy in patients with SLE leads to increased disease activity which is associated with adverse pregnancy outcomes [28]. Several studies have shown no significant risk of congenital malformations, retinopathy or ototoxicity in pregnancy [29–31].

## Effects of HCQ from malaria to autoimmune diseases

HCQ and CQ are weak bases with a positive charge and concentrate in acidic organelles such as endosomes, the Golgi apparatus, and lysosomes. Both molecules are unable to cross the cell membrane in their protonated form; however, the unprotonated portion enters the cell and is protonated inversely to pH; once protonated it is trapped in the acidic organelles [32].

Below, we present some modulating effects of HCQ that could explain its utility in different diseases.

### HCQ increases endoplasmic reticulum pH: effects on dyslipidemias

APS and SLE patients have higher cardiovascular morbidity and mortality than the general population, and hyperlipidemia control is essential to decrease it [33]. In a cohort of 264 SLE patients, treatment with 200–400 mg of HCQ daily resulted in a decrease in plasma cholesterol levels [34]. Furthermore, an increase in cholesterol after discontinuation of HCQ has been observed [35]. A systematic review that included 559 patients from eight studies found a reduction in low-density lipoprotein levels by using HCQ [36]. The exact effect of HCQ on the enzymes involved in cholesterol synthesis is unclear. However, CQ disables acetyl-coenzyme A, required in initial events of cholesterogenesis in the endoplasmic reticulum, suggesting that HCQ lowers cholesterol levels by increasing the pH in the endoplasmic reticulum [37]. Therefore, HCQ treatment could be co-adjuvant to conventional cardiovascular risk control tools in patients with APS and/or SLE.

### HCQ increases endosomal pH: effects on diabetes and viral infections

Case reports have described hypoglycemia in patients with RA treated with HCQ, with or without diabetes [38, 39]. Interestingly, the magnitude of the effect of HCQ on glucose levels has been sufficient to suppress the need for insulin [40]. The mechanism of HCQ on glucose metabolism may be related to inhibition of intracellular insulin degradation in human adipose tissue, by changes in lysosomal enzyme activity and intra-endosomal pH [41, 42].

In viral infections, it has been established that CQ inhibits pH-dependent steps in the replication of flavivirus, retrovirus, and coronavirus [32, 43]. In treating patients with HIV infection, 400 mg of HCQ has been included as an adjuvant for six months, as this resulted in an increase in the CD4-positive T lymphocyte count [44]. Furthermore, use of HCQ combined with hydroxycarbamide and didanosine for 48 weeks decreased viral load and increased CD4-positive T lymphocyte count [45]. HCQ could inhibit replication of the virus by increasing endosomal pH, which inhibits post-translational modifications of the gp120 glycoprotein, essential for the fusion of viral and cellular membranes [46]. Moreover, HCQ suppressed HIV replication by 50% as demonstrated in T lymphocytes obtained from pleural fluid from an individual infected with HIV and monocyte cell line 63_HIV_ [46]. Thus, through endosomal pH increase, HCQ seems to play an immunomodulatory role, which could lead to beneficial effects in patients with chronic inflammatory diseases such as obstetric APS without seeming to interfere with responses against pathogens [10].

Another important pathway modulated by HCQ is the pathway of Toll-like receptors (TLRs). TLRs in endosomes transmit signals that are regulated by pH. Among these TLRs are the TLR-4, -7, -8, and -9. In APS, TLRs are activated in the absence of pathogens and can lead to the production of pro-inflammatory cytokines and aPL [47]. Blocking in endosomal acidification by CQ inhibits TLR-3 and TLR-4 signal by attenuating the adapter-dependent signaling pathway that contains the Toll/IL-1 receptor domain (TIR), interferon-inducing and independently of myeloid differentiation primary response gene 88 (MyD88) [48–50]. Moreover, CQ decreases the activation of interferon response factors, nuclear factor kappa B (NF-kB) and activator protein 1 (AP-1) in monocyte-derived macrophages [48]. These events reduce the production of proinflammatory cytokines [51]. Finally, other pathways that CQ can inhibit involve the activation of p38MAPK by stress in the endoplasmic reticulum leading to increased proinflammatory cytokines [52, 53].

### HCQ increases lysosomal pH: effects in cancer

The effects of HCQ in cancer have been mainly related to the inhibition of autophagy and proliferation of cancer cells and induction of apoptosis [54, 55]. Autophagy is related to cell protection as a survival mechanism for tumor cells [56]. Preclinical studies indicate that combining HCQ with chemotherapy or radiotherapy can contribute to higher response rates of treatments against different types of cancer, even in advanced disease stages [57, 58]. For instance, HCQ and CQ reduced the proliferation of cells from human bladder cancer in a dose and time-dependent manner [54]. Besides, in metastatic kidney cancer cells, HCQ inhibited cell growth, oxygen consumption and mitochondrial respiration [59].

### HCQ effects in autoimmune diseases

In 1894, quinine was first used for the treatment of discoid lupus [6]. In this context, CQ was observed to interfere with phagocytosis due to increased lysosomal pH, causing an interruption in the presentation of self-antigens both in vitro and in vivo [60, 61]. The lysosomes contain acidic hydrolases that are active at a pH of 5 and are necessary for the degradation of proteins, nucleic acids, lipids, and carbohydrates [62]. For digestion of captured components after phagocytosis, a pH of 4.8 is required in these acidic organelles in contrast to the cytosol, which has a pH of 7.2 [62]. Interestingly, in an experimental autoimmune model of multiple sclerosis, inhibition of lysosomal proteases caused a reduction in the proinflammatory response and autoantigens presentation [63].

On the other hand, lysosomal pH is decreased in the immune response as it is required in antigenic processing, but the addition of compounds with tropism to lysosomes, such as CQ, inhibit catabolism and antigenic presentation to T lymphocytes by mouse macrophages [60]. HCQ modulates pH in lysosomes, which could alter the processing and presentation of self-antigens and, consequently, activation of macrophages and T lymphocytes in APS. Furthermore, HCQ modulates the activation of T lymphocytes by inhibiting calcium mobilization in the endoplasmic reticulum [64].

The efficacy of HCQ in the treatment of RA has been reported in randomized controlled clinical trials, in which it has been used in combination with methotrexate and sulfasalazine and is considered one of the safest and best-tolerated drugs licensed for the treatment of RA [65]. Furthermore, the efficacy of HCQ has especially been recognized in treating SLE patients both with and without secondary APS. [66]. Nowadays, HCQ is recommended for all SLE patients, especially during pregnancy and breastfeeding, where it is safe and effective in preventing disease flares and adverse pregnancy outcomes [67, 68]. HCQ use has been described in vascular and obstetric APS, especially in patients with SLE-related secondary APS. Nevertheless, its therapeutic potential has been linked to obstetric APS due to inflammation during placentation [14].

**Fig. 1:**
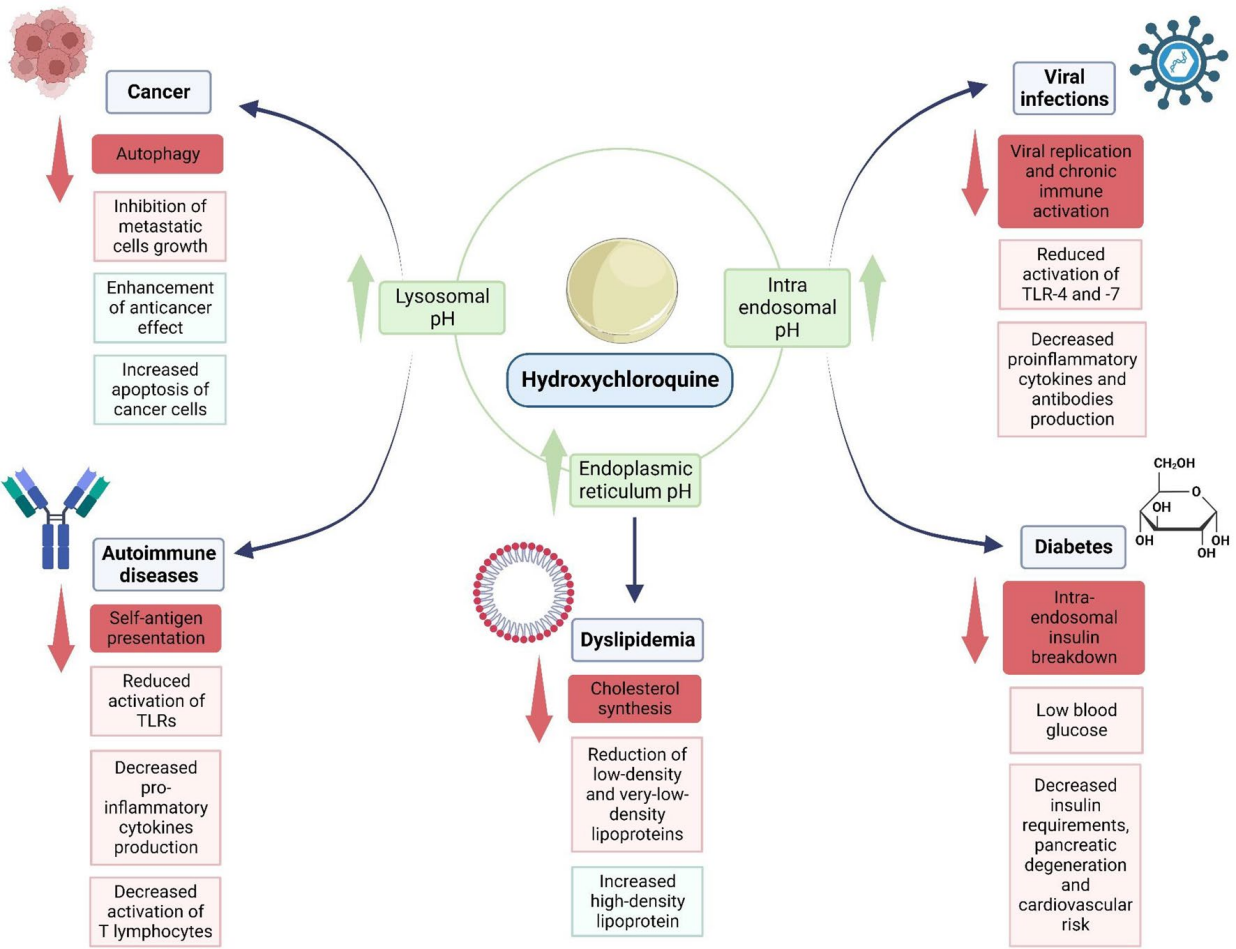
HCQ effects in different diseases mediated by pH modifications. HCQ induces beneficial effects in cancer, dyslipidemia, viral infections, diabetes and autoimmune diseases through pH modifications. Abbreviations. TLRs, toll-like receptors. This figure was created with BioRender.com

In summary, the modulating effects of HCQ in several conditions have been associated with increased pH in different cellular compartments such as endoplasmic reticulum, endosomes and lysosomes reducing TLR activation, increasing apoptosis of cancer cells and decreasing pro-inflammatory cytokines which leads to positive effects in diabetes, dyslipidemias, cancer, viral infections, and autoimmune diseases such as APS, SLE and RA. Underlying mechanisms of HCQ involved in these processes are described in Fig. 1*.* In the remaining part of this review, we will summarize published studies describing these and other modulating effects of HCQ in APS, especially in refractory obstetric APS.

## HCQ possible modulating effects in obstetric APS

APS-related pregnancy morbidity is associated with the presence of aPL that induces complement activation, cytokine production and placental dysfunction [69, 70]. Therefore, obstetric APS is mainly characterized by a proinflammatory state during placentation related to reduced trophoblast invasion and impaired spiral artery remodeling at early pregnancy stages [10]. Moreover, aß2GP1 antibodies have been identified as key pathogenic factors due to their effects on the maternal–fetal interface [70]. In APS, HCQ can modulate pH by similar pathways as described above [71, 72]. Furthermore, other mechanisms are described in patients with APS, as HCQ inhibits complement activation, reduces production of tissue factor (TF), disintegrates binding of aß2GP1 to ß2GP1 and complexes of phospholipid bilayers, inhibits production of tumor necrosis factor α (TNFα) in monocytes, restores anticoagulant action of annexin A5, reduces trophoblast alteration, fetal death, placental insufficiency, thrombosis, endothelial dysfunction and decreased LA levels *(*Table 1*)*. Furthermore, our Reproduction research group found that both aPL from primary and secondary refractory APS patients induced endothelial dysfunction in vitro*,* and HCQ restored endothelial function in cells treated with aPL from women with refractory primary obstetric APS [73].

**Table 1:** In vitro and in vivo effects of HCQ in antiphospholipid syndrome

APS classification	Clinical manifestation	Source and dose of aPL	Number of patients	Study model	HCQ dosage	Time of exposition	Mechanism of modulation	References
Secondary APS	PM and VT	LA, aCL and aß2GP1 (100 μg/ml)	20 (14 women and 6 men)	In vitro (monocytes)	1 μM	6 h and 15 m	Inhibition of TNFα production (without modulating effects of some aß2GP1)	[87]
Not specified	PM and VT	Monoclonal antibody (0.1 mg/ml) and aPL-NS (0.5 mg/ml)	3 (gender not specified)	In vitro (monocytes and phospholipid bilayer)	> 1 μg/ml	0–4 h	Reduction of aPL/PL/ß2GP1 complex formation	[76]
Catastrophic APS	PM and VT	LA, aCL and aβGP1 (0.5 mg/ml)	3 (2 women and 1 men)	In vitro (endothelium, trophoblast, and phospholipid bilayer)	1 μg/ml and 1 mg/ml	0–4 h	Restoration of the anticoagulant action of Annexin A5	[88]
NA	NA	Monoclonal antibody aß2GP1 (20 μg/ml)	NA (antibodies produced in mice)	In vitro (trophoblast)	1 μg/ml	72 h	Reversal of IL-6 inhibition and partial modulation of impaired migration. It does not modulate the secretion of proinflammatory cytokines such as IL-8 and IL-1β	[89]
Not specified	PM and VT	LA, aCL and aß2GP1 (4.2 mg/mouse)	5 (women)	In vitro (syncytiotrophoblast)	0.1, 0.2, 0.5 and 1 μg/ml^−1^	48 h	Restoration of trophoblastic fusion by a decrease of TLR-4	[90]
Primary APS	VT	Monoclonal antibody aß2GP1 (0.1 mg/ml)	1 woman	In vitro (phospholipid vesicles)	0,01- 0,5 mg/ml	0–250 min	Reduction of aPL/PL/ß2GP1 complex formation	[91]
Refractory primary/secondary, and non-refractory APS (thrombotic or obstetric)	PM and VT	LA, aCL and aß2GP1	21 women	In vitro (endothelium)	1 μg/ml	1 h	Partial restoration of endothelial function altered by aPL from patients with refractory primary obstetric APS	[73]
Primary APS	PM and VT	aß2GP1 (100 μg/mouse)	7 (5 women and 2 men)	In vivo (C57BL/6 mice)	In vivo (200 μg/mouse)	Insertion with a micro-osmotic pump on day 8 of gestation	Prevention of fetal death, placental insufficiency, cortical development abnormality, decreases C5a levels, and C´ activation. It does not inhibit the binding of aPL to brain tissue but impede their pathological effect	[69]
				Ex vivo (mouse tissue)	Ex vivo (20–50 and 100 ng/ml)			
Primary APS	Not specified	aCL and aß2GP1	6 (gender not specified)	In vivo (C57BL/6 mice)	In vivo 12 μg/g/day	7 days	Reduction of thrombosis by decreased TF and modulation of endothelial dysfunction and p-eNOS expression	[92]
				Ex vivo (mouse tissue mesenteric arteries)	Ex vivo or in vitro 1 and 10 mg/ml			
				In vitro (human aortic endothelial cells)				
Not specified	PM and VT	LA, aCL and aß2GP1	22 (20 women and 2 men)	Prospective observational study	200 mg/day	3 months	Reduction of TF levels. It did not affect Annexin A5 activity, on aß2GP1, thromboelastography, on CRP, C´, and VEGF levels	[93]
Primary APS	PM and VT	LA, aCL and aß2GP1	114 (100 women and 14 men)	Retrospective study	Not specified	12 months	Reduction of aCL, aß2GP1, and thrombotic events	[94]

*APS* antiphospholipid syndrome, *NS* not specified, *NA* not applicable, *PM* pregnancy morbidity, *VT* vascular thrombosis, *LA* lupus anticoagulant, *aCL* anti-cardiolipin antibodies, *aß2GP1* anti-ß2 glycoprotein 1 antibodies, *μM* micromolar, *μg* micrograms, *ml* milliliters, *mg* milligrams, *ng* nanogram, *g* grams, *h* hours, *m* minutes, *TNFα* tumor necrosis factor alpha, *PL* phospholipids, *ß2GP1* ß2 glycoprotein 1, *IL* interleukin, *C´* complement, *TLR-4* Toll-like receptor 4, *TF* tissue factor, *p-eNOS* phosphorylated endothelial nitric oxide synthase, *CRP* C-reactive protein, *VEGF* vascular endothelial growth factor

HCQ also plays a role in the disintegration of autoimmune complexes, since, in the antigen–antibody reaction, the effect of pH on the equilibrium constant is characterized by a curve between pH 6.5 and 8.4 [74]. At different values, the equilibrium constant is 100 times lower, since these extreme conditions generate conformational changes in the antibody that alter its complementarity with the antigen [74]. Depending on the type of antibody, it will have a specific pH at which it can bind to the antigen in different intracellular compartments [75]. Interestingly, HCQ could avoid binding of aPL to the plasma membrane or cause breakdown of aPL complexes, but the pH values at which these aPL bind to antigens are unknown [76, 77]. The disintegration of the complexes by HCQ prevents the different cascades triggered by the aPL such as the decrease of AnxA5, complement activation, decrease of endothelial nitric oxide synthase, TF production, thrombosis, and placental dysfunction. Furthermore, HCQ inhibits translocation of the catalytic subunit of NADPH oxidase 2 (NOX2) from the cell membrane to the endosome, consequently blocking the production of endosomal superoxide mediated by aPL [78]. Finally, HCQ effects on TLR could inhibit pro-inflammatory responses following aPL and ß2GP1 interaction in the trophoblast [79, 80].

## Clinical effects of HCQ in obstetric APS

The conventional treatment of obstetric complications in APS patients is based on the daily use of low-dose aspirin (75–100 mg) and low molecular weight heparin (LMWH) [14]. Nevertheless, patients with refractory obstetric APS do not respond to this conventional therapy. These patients most often have higher aPL titers, concomitant clinical manifestations of thrombosis, and other autoimmune diseases [81].

Five retrospective studies showed an improvement in live birth rate in women with persistent aPL positivity when HCQ was added to conventional treatment regimens [82]. In 87 patients with refractory primary obstetric APS, women treated with 60 mg of enoxaparin, low-dose aspirin and 400 mg of HCQ had a live birth rate of 97.1% (67/69) compared with 62% (20/30) in those who did not receive HCQ. Moreover, a low prevalence of other pregnancy complications in APS patients treated with HCQ was reported [83]. Interestingly, in a cohort of aPL-positive women, pregnancy failures occurred in 30.9% under conventional treatment with low-dose aspirin and LMWH. A reduction of pregnancy failures to 22% was observed in the subsequent pregnancies of those refractory patients after adding HCQ [84]. Hooper et al. described an increase in the achievement of live births and better maternal and neonatal outcomes after adding HCQ in patients with refractory obstetric APS [15].

**Fig. 2:**
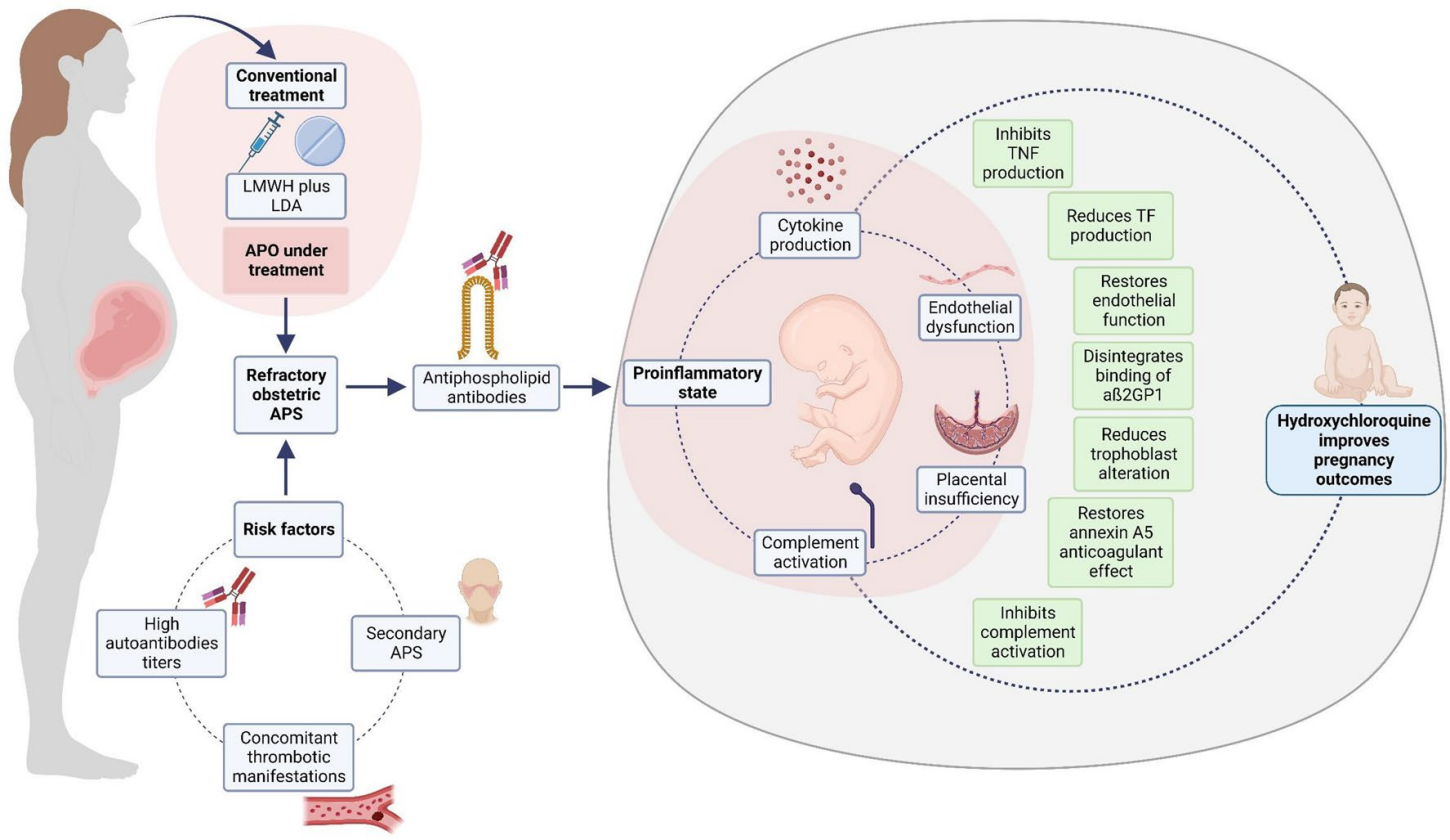
HCQ benefic effects in refractory obstetric APS. HCQ improves pregnancy outcomes in patients with refractory obstetric APS by restoring endothelial function, decreasing complement and cytokine production, and preventing placental damage. *LMWH* low molecular weight heparin, *LDA* low-dose aspirin, *APO* adverse pregnancy outcomes, *APS* antiphospholipid syndrome, *TNF* tumor necrosis factor, *TF* tissue factor, *aß2GP1* anti-ß2 glycoprotein 1 antibodies. This figure was created with BioRender.com

In different reports, HCQ modulated pathological effects generated by aPL in men and women with primary APS, secondary APS, catastrophic APS, and clinical manifestations of thrombosis and pregnancy morbidity. The beneficial effects of HCQ and their underlying mechanisms in refractory obstetric APS are summarized in Fig. 2*.* Some results are controversial between studies and, lack of stratification between different aPL and clinical manifestations has made it difficult to understand the exact mechanism of action of HCQ in obstetric APS. It is possible that, according to these characteristics, different doses, and periods of exposure to the drug are required. Furthermore, the need to start treatment with HCQ in the preconception period rather than the first trimester and pharmacokinetic changes due to pregnancy should be addressed in the treatment schemes for APS patients.

These results suggested that HCQ might be used in patients with refractory obstetric APS, and we encourage its implementation in clinical practice. However, available data mainly come from small retrospective studies; hence, future prospective studies and randomized clinical trials with a larger population of patients, including primary and secondary APS, are required to demonstrate which patients would benefit most and set the best suitable treatment scheme. Currently, efforts assessing the HCQ use in patients with obstetric APS occur through the HYPATIA and HIBISCUS clinical trials [85, 86]. Finally, the effects of usually added treatments in obstetric refractory APS such as glucocorticoids and intravenous immunoglobulin should be addressed in future studies.

## Conclusion

In this review, we described distinct positive effects of HCQ in different pathologies as well as in obstetric APS. These results suggest that HCQ should be a highly valuable addition to the treatment of refractory obstetric APS. Although most of the in vitro information is based on studies conducted with CQ, HCQ is the more favorable alternative due to lower risks of adverse effects, lower dosage requirements, and the greater identified effectiveness in some essays. Nevertheless, HCQ-related effects in different subgroups of APS patients remain unclear. Thus, we encourage a detailed classification of APS subgroups in further studies.

## Declarations

### Disclaimer

No part of the review, including its graphics, are copied or published elsewhere in whole or in part.
